# Pharmacometabolomics of treatment response to antihypertensive drugs: a systematic review

**DOI:** 10.1097/HJH.0000000000004355

**Published:** 2026-06-20

**Authors:** Elahe Zare Borzeshi, Merys Valdez, Alida Kindt, Banafsheh Arshi, Elham Memarian, Thomas Hankemeier, Dipak Kotecha, Rick Grobbee, Jonathan E. Knikman

**Affiliations:** a Department of Global Public Health & Bioethics, Julius Center for Health Sciences and Primary Care, University Medical Center Utrecht, Utrecht; b Metabolomics and Analytics Centre, Leiden Academic Centre for Drug Research (LACDR), Leiden University, Leiden; c Institute for Risk Assessment Sciences, Utrecht University, Utrecht; d Department of Epidemiology, CAPHRI School for Public Health and Primary Care, Faculty of Health, Medicine and Life Sciences, Maastricht University, Maastricht, the Netherlands; e Department of Cardiovascular Sciences, University of Birmingham; f NIHR Birmingham Biomedical Research Centre, University Hospitals Birmingham NHS Foundation Trust, Birmingham, UK; g Julius Center for Health Sciences and Primary Care, University Medical Center Utrecht, Utrecht, The Netherlands; h https://www.hypermarker.eu/

**Keywords:** antihypertensive drugs, hypertension, metabolomics, pharmacometabolomics

## Abstract

Hypertension remains a prevalent, modifiable risk factor for cardiovascular disease, with many patients failing to achieve optimal blood pressure (BP) control despite existing therapies. Pharmacometabolomics offers promise by identifying metabolic biomarkers linked to drug response and holds potential for individualizing antihypertensive treatment. We conducted a systematic review of studies from 2013 to 2023, screening 2577 articles and including five that investigated metabolomics-based biomarkers for antihypertensive drug response in cases of primary hypertension. In total, 46 metabolites were significantly associated with BP responses to diuretics, beta-blockers, and ACE inhibitors or angiotensin receptor blockers. Key predictive metabolites included arachidonic acid, sphingosine-1-phosphate, 2-oxoglutarate, and arachidonoyl-carnitine, affecting responses across fatty acid metabolism, sphingolipid metabolism, the TCA cycle, and amino acid metabolism. This review highlights the potential of pharmacometabolomics to uncover metabolites and pathways associated with individual BP response to antihypertensive drugs. Further validation in diverse populations, drug classes, and combination treatments is needed.

## Graphical abstract

**Figure:**
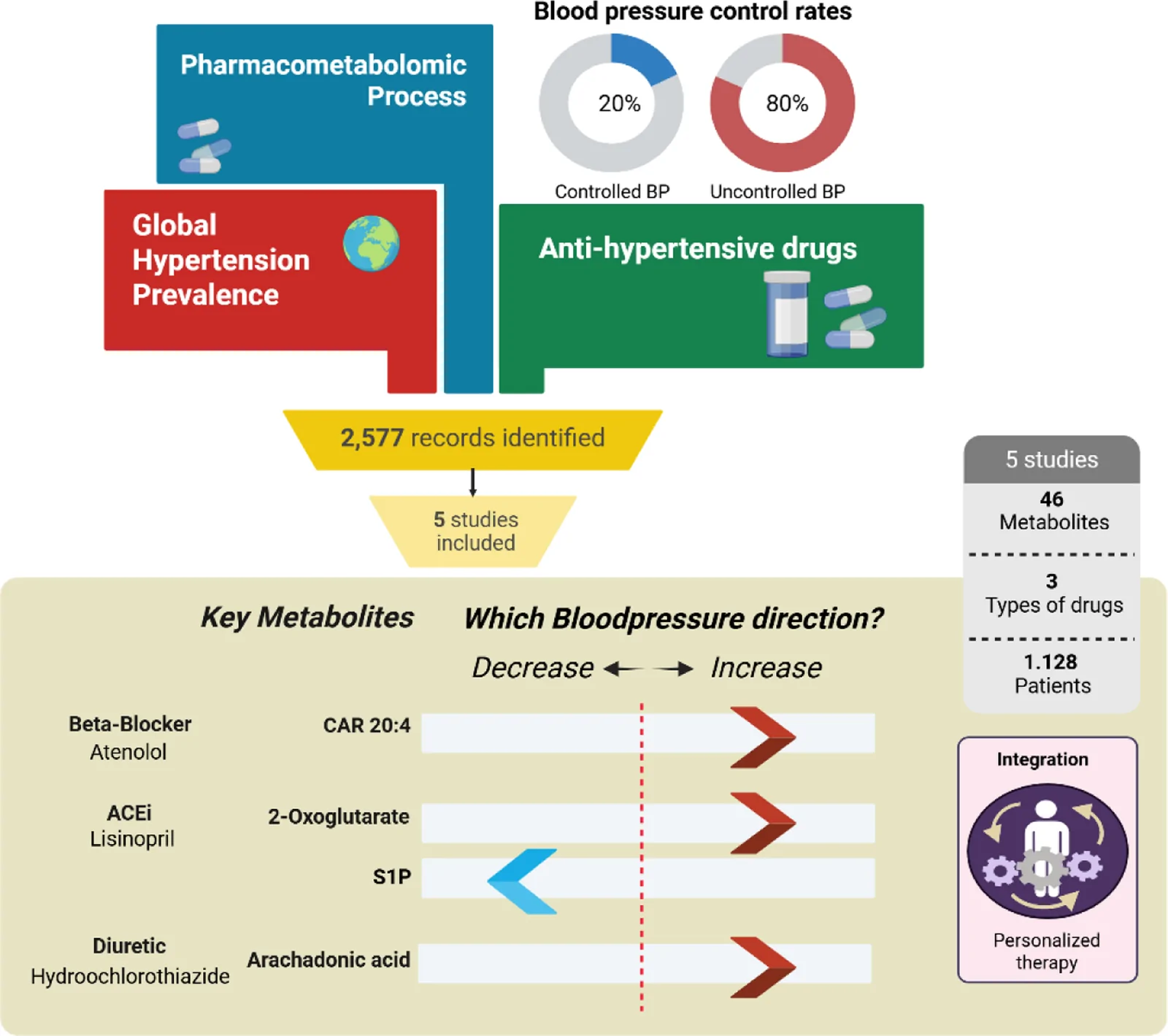

## INTRODUCTION

Hypertension affects more than a billion people worldwide, is a major uncorrected risk factor for stroke, myocardial infarction and vascular dementia, and has a profound economic burden [1]. Currently, only 20% of adults with hypertension manage to maintain adequate blood pressure (BP) control [2,3].

Antihypertensive drugs remain the cornerstone of BP control by targeting the renin angiotensin aldosterone system, the sympathetic nervous system (SNS) and calcium-signaling pathways to reduce vascular resistance, modulate cardiac output and promote vasodilation [4]. Despite the availability of multiple classes of efficacious BP-lowering medications, treatment control rates remain suboptimal. This gap underscores the need for improved therapeutic strategies beyond conventional treatments protocols. Personalized medicine offers a promising approach to optimize antihypertensive therapy by tailoring treatment to individual patient characteristics or biomarkers. Realizing this potential requires study designs capable of accounting for the substantial inter-individual variability in BP response observed in clinical practice. In a randomized crossover trial conducted under controlled conditions, four commonly used antihypertensive agents such as lisinopril (angiotensin-converting enzyme inhibitor), candesartan (angiotensin receptor blocker), hydrochlorothiazide (thiazide diuretic) and amlodipine (calcium channel blocker), were compared within the same individuals [5]. The study revealed significant variability in BP response across drug classes, reinforcing the potential for individualized treatment strategies. Notably, personalization based on patient response achieved an additional average reduction of 4.4 mmHg in SBP compared to nontailored (fixed) therapy, suggesting clinically meaningful benefits [5]. However, despite more than two decades research in biomarkers, genetics and pharmacogenomics research, few predictors have proven clinically actionable predictors to inform antihypertensive drug selection [6–8].

Pharmacometabolomics examines the metabolome, the comprehensive profile of endogenous small molecules, to inform potential approaches to personalizing antihypertensive therapy by revealing the biochemical pathways underlying BP regulation and individual drug responses. Integrating metabolic biomarkers, often measured in biomatrices such as blood, with patient-specific physiological and environmental factors may enhance treatment efficacy and help explain phenotypic variability in drug response [5,9–12]. Although both targeted and untargeted metabolomic approaches in blood samples are still relatively new in the context of antihypertensive pharmacotherapy, they offer complementary information: targeted assays enable precise quantification of predefined metabolites in candidate pathways implicated in BP regulation, whereas untargeted profiling provides broad coverage to detect a wide range of known and previously uncharacterized metabolites and pathways that may influence treatment response. This systematic review explores the current evidence to identify metabolites and their proposed biological pathways that could be used to predict the response of common blood pressure lowering treatments in primary hypertension patients.

## MATERIALS AND METHODS

This systematic review protocol was prospectively registered with PROSPERO (registration number: CRD42024501059). Ethical approval was not required as data were collected from previously published studies, which involved anonymised patient data. This systematic review was conducted as part of the HYPERMARKER project, which aims to explore the clinical utility of pharmacometabolomics in assisting treatment decisions for antihypertensive therapy. The programme is funded by the European Union (EU) and UK Research and Innovation (UKRI).

### Search strategy and eligibility criteria

In this systematic review, we searched PubMed/MEDLINE, EMBASE, Scopus, Web of science for studies reporting on metabolomics of hypertension treatment, covering the period from January 2013 and December 2023, with an updated search conducted in June 2025 to ensure no new eligible studies had been published. This study followed the guidelines of Preferred Reporting Items for Systematic Reviews and Meta-Analyses (PRISMA) [13]. The search strategy included terms related to metabolomics, antihypertensive drug classes, and primary hypertension. A complete search strategy and including exact search terms and Boolean operators and filters, are provided in Supplementary Table S1. The following publication types were included in the search strategy: observational study, randomised controlled trial, and clinical trial. We also limited our search to human subjects and articles available in English. Additional grey literature was identified through supplementary sources.

The analysis was restricted to individuals with primary hypertension. All contributing studies explicitly excluded participants with suspected or confirmed secondary causes of hypertension, including renal and endocrine conditions such as primary aldosteronism, through protocol-specified eligibility criteria. Therefore, secondary hypertension was not within the scope of the present investigation. Studies focusing exclusively on severe hypertension (SBP ≥180 mmHg and/or DBP ≥110 mmHg) were excluded. This was done to reduce bias from potential confounders such as acute end-organ damage, emergency management protocols, or polypharmacy, all of which may impact metabolomic profiles independently of antihypertensive drug response. Studies including participants treated with multiple antihypertensive drugs were included if metabolite associations could be attributed to specific drug classes or were analysed separately if such differentiation was reported. Studies in which results could not be distinguished by antihypertensive class were excluded from class-specific analyses.

### Outcomes

The primary outcomes were the identification of specific metabolites predictive of antihypertensive drug response and their association with changes in BP in primary hypertension patients. Secondary outcomes included characterization of biological pathways associated with metabolite responses.

### Data collection and risk of bias assessment

Articles were excluded based on irrelevance to pharmacometabolomics, lack of clear association between metabolites and BP outcomes, absence of specific metabolite identification, and inadequate quality annotation.

Initial title screening was performed by one reviewer (EZ), followed by independent abstract reviews by two reviewers (EZ, MV) using a predefined eligibility checklist. Full-text articles deemed potentially eligible were retrieved and independently evaluated for final inclusion by two reviewers (EZ, MV). Discrepancies were resolved through consensus discussions involving a third reviewer (JK). Data extraction was conducted by one reviewer (EZ) and verified independently by additional reviewers (MV, JK) for accuracy and completeness. Extracted data included drug class, metabolites, main findings, author, study year, study design, and quality assessment score. Quality and risk of bias in the included studies were assessed using standardised checklists appropriate to study design: the Strengthening the Reporting of Observational Studies in Epidemiology (STROBE) checklist for observational studies and the Consolidated Standards of Reporting Trials (CONSORT) checklist for randomized controlled trials (RCTs) [14,15]. Each study was rated for potential biases in selection, outcome measurement, intervention consistency, and completeness of reported data.

### Data synthesis

Data from included studies were synthesised descriptively, with metabolites categorised by antihypertensive drug classes (diuretics, beta-blockers, ACE inhibitors). A qualitative synthesis summarised metabolite associations with BP response. Pathway analyses identified common biochemical pathways associated with significant metabolites. Results were presented using descriptive tables summarizing metabolite associations and potential mechanisms linked to antihypertensive drug efficacy.

### Data visualization

A Sankey plot was generated using R version 4.2.1, with the visualization created through the ggplot2 package. The plot illustrates the relationships between metabolites and metabolic pathways, based on connections identified in the existing literature.

## RESULTS

**FIGURE 1:**
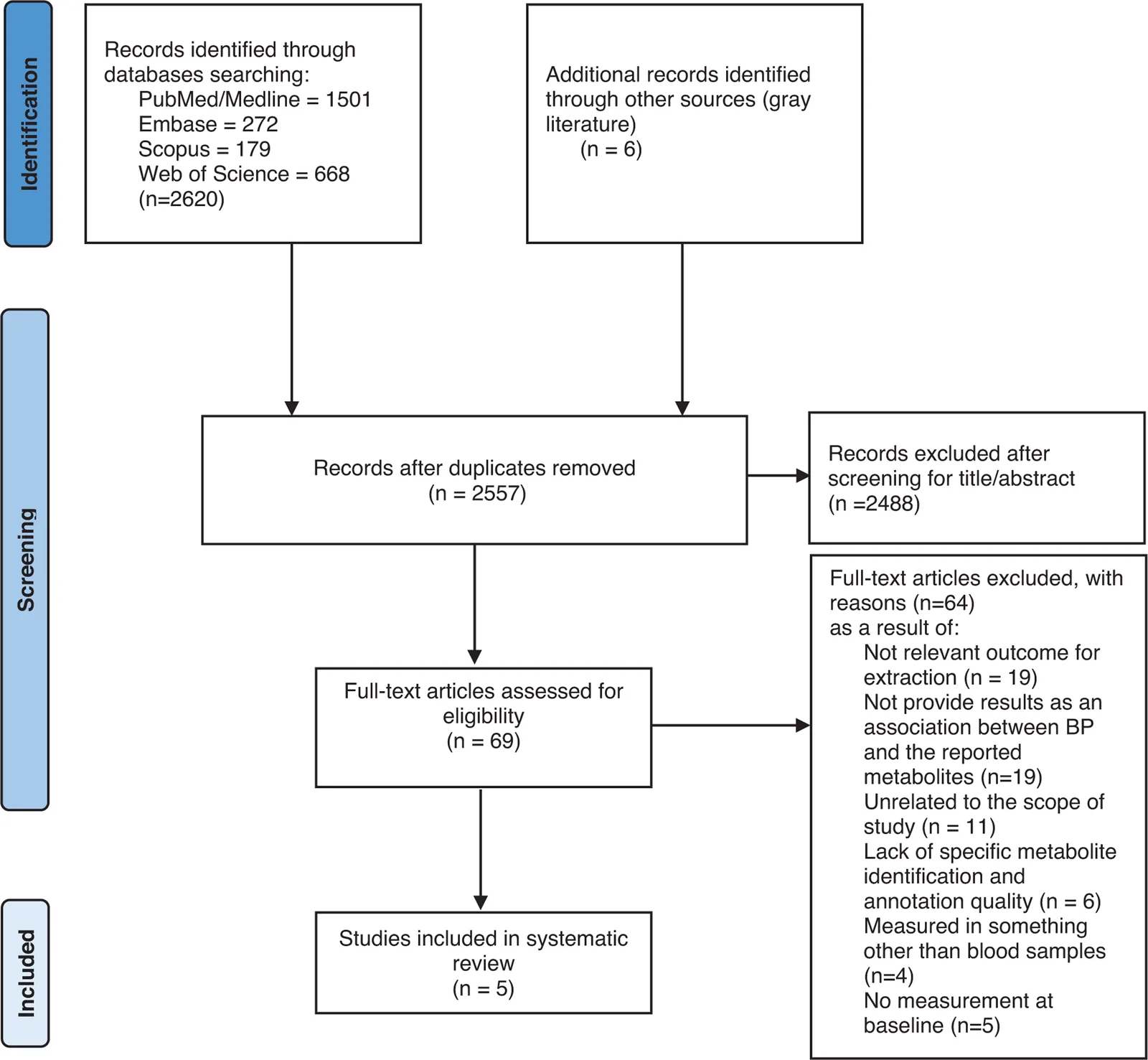
PRISMA flow chart illustrating the selection process of the studies.

A total of 2577 articles were screened, of which 69 were evaluated at full text and 5 studies including 875 participants were eligible for analysis (Fig. 1).

Comprehensive descriptions of the included studies are available in the original publications [16–20]. Eligible studies included adults aged 17–89 years diagnosed with mild-to-moderate hypertension, defined as a SBP of 140–179 mmHg and/or a DBP of 90–109 mmHg, in accordance with international guidelines [2]. Typical exclusion criteria included severe or secondary hypertension, diabetes mellitus, pregnancy, and significant cardiovascular, renal, or hepatic diseases. Detailed methods of metabolomics, BP assessment, and other characteristics of the included studies are presented in Table 1.

**TABLE 1:** Summary of included studies

Author (Year)	No. patients	Patient population	Patient type/Inclusion criteria	Relevant exclusion factors	Study design	Metabolomics measurement method	Targeted/Untargeted	BP assessment method
Rotroff *et al.* (2015) [21]	433	White (*n* = 251), Black (*n* = 192)	Adults aged 17–65, mild-to-moderate hypertension, enrolled in PEAR study	Cardiovascular, renal/hepatic disease, diabetes	Observational	GC-TOF/MS	Untargeted	Home BP monitoring
Weng *et al.* (2016) [24]	224	White	Adults aged 17–65, mild-to-moderate hypertension	Severe hypertension, diabetes, pregnancy, renal diseases	Observational	LC-MS	Targeted	Composite BP (office, home, ambulatory)
Shahin *et al.* (2017) [22]	123	White	Adults aged 17–65, mild-to-moderate hypertension	Cardiovascular, renal/hepatic disease, diabetes	Observational	UHPLC-MS, lipidomics	Both	Office, home, ambulatory BP
Sonn *et al.* (2019) [20]	45	White	Adults aged 18–89 years, uncontrolled hypertension	Not explicitly stated	Observational	UHPLC-MS, lipidomics	Targeted	Automated cuff, office BP
Huang *et al.* (2022) [23]	50	Asian	Adults ≥25 years, mild-to-moderate essential hypertension	Severe/secondary hypertension, chronic diseases, pregnancy	Observational	UPLC-MS/MS, FIA-MS/MS	Targeted	Office, ambulatory BP

BP, blood pressure; FIA, flow injection analysis; GC, gas chromatography; LC, liquid chromatography; MS, mass spectrometry; PEAR, Pharmacogenomic Evaluation of Antihypertensive Responses; TOF, time-of-flight; UHPLC, ultra-high performance liquid chromatography; UPLC, ultra-performance liquid chromatography.

### Risk of bias

The risk of bias assessment revealed variability in methodological quality among the studies included. Given that most studies were secondary analyses of observational they inherently carried risks of bias, particularly selection and ascertainment biases. Despite the studies employing standardised methods and clearly reporting key outcomes, none provided comprehensive protocols explicitly addressing the potential sources of selection or performance bias. As a result, the overall risk of bias for the studies included ranged from moderate to high. The detailed quality assessment scores and domain-specific bias ratings for each study are provided in Table 2.

**TABLE 2:** Metabolites associated with blood pressure response

Drug class	Metabolite	Association with BP-response	Main findings	First author	Study year	Study design	Quality assessment score^d^	Ref.
Diuretics	Palmitoleic acid	Positive^b^	• Baseline level associated with HDBP response	Rotroff *et al.*	2015	Prospective cohort	9	[21]
	Salicylic acid							
	Arachidonic acid		• Baseline level associated with HDBP response in “white” patients					
	223548 (Unknown)	Positive/ negative	• Baseline level positively associated with HDBP response in “white” patients • Baseline level negatively associated with HDBP response in “black” patients					
	Glycolic acid	Not specified^b^	• Significantly associated with hydrochlorothiazide blood pressure response (the association of with BP response was not specified in terms of positive or negative) • Sphingolipid metabolism pathway might be involved in the long-term mechanism underlying HCTZ response.	Shahin *et al.*^a^	2016, 2017	Prospective cohort	9	[22,25]
	Fumaric acid							
	Arachadonic acid							
	Caprylic acid							
	Dodecanol							
	Iminodiacetic acid							
	Trihydroxypyrazine							
	Pyrazine 2,5 dihydroxy							
	2 hydroxyvaleric acid							
	Dihydroabietic acid							
	Phytol							
	2 hydroxybutanoic acid							
	Arabinose							
	Aspartate	Positive	• Baseline levels associated with SBP response • High abundance of the positively associated metabolites was related to poor BP response to HCTZ (and vice versa) • High abundance of the negatively associated metabolites was related to good BP response to HCTZ (and *vice versa*)	Huang *et al.*	2022	Prospective Cohort	9	[23]
	Glutamate							
	Lysophosphatidylcholine C16 : 0							
	Lysophosphatidylcholine C20 : 3							
	Sphingomyelin C24 : 1	Negativeb						
	Lysophosphatidylcholine C20 : 3	Positive	• Baseline levels associated with DBP response					
Beta-blockers	O-acetylserine	Negative	• Baseline levels associated with HDBP response	Rotroff *et al.*	2015	Prospective Cohort	9	[21]
	6-deoxyglucitol	Positive						
	216043 (Unknown)							
	Fucose+rhamnose							
	Beta-mannosylgelycerate							
	5-Methyloxythryptamine	Negative	• Baseline level associated with HDBP response in “white” patients					
	340257 (Unknown)	Negative	• Baseline level associated with HDBP response in “black” patients					
	2,4-diaminobutyric acid							
	213253 (Unknown)	Positive						
	Arabitol							
	228377 (Unknown)							
	470983 (Unknown)							
	483390 (Unknown)							
	Arachidonoyl-carnitine CAR (20 : 4)	Increased	• Presence of CAR (20 : 4) was significantly associated with poorer BP-lowering efficacy	Weng *et al.*	2016	Prospective Cohort	9	[24]
ACEi	2-Oxoglutarate	Decreased^b^	• All metabolites and their abundance refer to responders (e.g., decreased levels of 2-oxoglutarate were associated with responders) • After absolute quantification of metabolites only 2-oxoglutarate remained significantly associated to BP response.	Sonn *et al.*	2019	Cohort	9	[20]
	Indole-3-acetaldehyde^c^							
	Inosine^c^	Increased^b^						
	Sphingosine^c^							
	Sphingosine 1-phosphate^c^							
	9-oxononanoic acid^c^							
	Butanoic acid^c^							
	Gamma-glutamylputrescine^c^							
	N5-methyglutamine^c^							
	Gamma-glutamygamma-aminobutyrate^c^							

ACEi, angiotensin-converting enzyme inhibitor; BP, blood pressure; HCTZ, hydrochlorothiazide; HDBP, home DBP; MetID, Metabolite Identification.

a Using the standard checklists for quality assessment; STROBE (Strengthening the Reporting of Observational Studies) for observational studies and CONSORT (Consolidated Standards of Reporting Trails) for randomised controlled trials (RCTs).

b Positive/increased values indicate that a higher concentration of these metabolites at baseline is associated with blood pressure, while negative/decreased values represent a lower concentration. Metabolites labelled as “not specified” were noted as significantly related without a specific direction of their concentration change.

c The relative quantitation of peak intensities for these metabolites revealed a significant differential expression between responders and nonresponders (*P* < 0.05). However, no significant differences were observed in absolute quantitation analysis.

^d^The research article identified in our systematic review presents results that were previously reported in an earlier publication by the same authors.

### Metabolites correlated with blood pressure response

A total of 46 metabolites were identified as predictive biomarkers for changes in BP in response to antihypertensive treatments. These metabolites were found to vary across different drug classes, with data synthesis suitable for diuretics, beta-blockers, and angiotensin-converting enzyme inhibitors (ACEi).

Studies were considered suitable for data synthesis if they reported specific baseline metabolite associations with BP response to clearly defined antihypertensive drug classes, provided adequate quantitative data, and met all predefined inclusion criteria.

Among the five studies assessed, three evaluated thiazide diuretics, one evaluated ACE inhibitors, and two assessed beta-blockers; no studies of calcium channel blockers meeting all inclusion criteria were identified.

#### Diuretics

Three articles [21–23] (*n* = 632 participants) reported the association of baseline metabolites with BP response to thiazide diuretic therapy. Across these studies, thiazide diuretic treatment produced modest-to-moderate BP reductions: in Huang *et al.* [23], hydrochlorothiazide reduced office BP by −18.6/−10.2 mmHg (SBP/DBP) and 24-h BP by −13.2/−7.1 mmHg, while in Shahin *et al.* [22] and Rotroff *et al.* [21], hydrochlorothiazide-based regimens were associated with average composite or home BP changes of approximately −11/−6 and −4 to −7 mmHg, respectively. Within this overall BP-lowering range, metabolites such as arachidonic acid and related lipid species measured at baseline were associated with BP responses, typically only by a few mmHg, which may help explain the inconsistent reporting of association direction across studies and BP phenotypes. Arachidonic acid was identified in two studies [21,22] of thiazide diuretics as significantly associated with changes in both SBP and DBP (false discovery rate < 0.05, *P* < 0.01, and q-values ranging from 0.036 to 0.067). Other identified metabolites included lipids such as sphingomyelins (e.g., SM C24 : 1, N24 : 2), phosphatidylcholines (e.g., PC ae C34 : 1, C34 : 3), and lysophosphatidylcholines (e.g., lysoPC C16 : 0, C20 : 3); organic acids, including uric acid, salicylic acid, glycolic acid, and ribonic acid; and amino acids, including aspartate, glutamate, and histidine. These metabolites were associated with baseline levels predictive of BP response to hydrochlorothiazide, with several findings reaching statistical thresholds of false discovery rate < 0.05 and replicated across cohorts. Association directions are summarized in Table 2.

#### Beta-blockers

Two articles [21,24,25] (*n* = 451 participants) reported baseline metabolomic associations with BP response to atenolol. On average, atenolol therapy lowered composite BP by −12.7/−10.4 mmHg (SBP/DBP) in the included cohort. Metabolites such as O-acetylserine, 2,4-diaminobutyric acid, and 5-methoxytryptamine were reported to be associated with poorer BP response, while sugar alcohols such as arabitol and 6-deoxyglucitol were associated with improved response (false discovery rate q-values < 0.2). In a separate analysis, the presence of arachidonoyl-carnitine (CAR 20 : 4) was linked to attenuated SBP and DBP-lowering efficacy, increased fasting glucose, and reduced HDL-C following atenolol therapy (*P* = 0.006 for systolic BP, *P* = 0.002 for diastolic BP). Quantitatively, the presence of CAR 20 : 4 was associated with approximately 3 mmHg less SBP, and 2 mmHg less DBP reduction compared with individuals without detectable CAR 20 : 4, indicating that the main metabolites of interest influenced BP response within a relatively narrow range around the mean drug effect. This modest effect size may partly explain why some associations appear as increases and others as decreases across different studies and analytic approaches.

Details on association direction are provided in Table 2.

#### Angiotensin-converting enzyme inhibitor

One article [20] (*n* = 45 participants) reported the association of baseline metabolites with BP response to ACEi therapy. In this study of lisinopril, BP response was defined as at least 10% decline in SBP from baseline to follow-up, and patients were classified as responders or nonresponders based on this threshold. Decreased levels of 2-oxoglutarate were associated with responders, while elevated levels of sphingosine, sphingosine 1-phosphate, butanoic acid, and N5-methylglutamine were also identified in those who responded to treatment. These metabolites implicate pathways related to amino acid, lipid, and short-chain fatty acid metabolism in modulating BP response to renin-angiotensin system inhibitors. These associations were identified with statistical significance (*P* < 0.05). Though findings are limited to a single cohort, they highlight potential biomarker candidates for future studies. Overall, these data suggest that, similar to other drug classes, metabolite profiles in ACEi-treated patients, which may reflect differences in the underlying biological systems that modulate the BP response to ACEi therapy, may be associated with interindividual differences in BP response around an underlying modest-to-moderate treatment effect, Because these metabolite-associated differences in BP response are relatively small and were derived from a binary responder definition in a single, small cohort, even minor differences in study design, BP phenotype, or response thresholds in other populations are likely to yield heterogeneous and sometimes apparently conflicting results. A detailed summary of identified metabolites and their associations with BP responses across the included studies is provided in Table 2.

Overall, these data suggest that, similar to other drug classes, metabolite profiles in ACEi-treated patients may be associated with interindividual differences in BP response around an underlying modest-to-moderate treatment effect. Because these metabolite-associated differences in BP response are relatively small and were derived from a binary responder definition in a single, small cohort, even minor differences in study design, BP phenotype, or response thresholds in other populations are likely to yield heterogeneous and sometimes apparently conflicting results.

#### Pathway analysis for identified metabolites

Pathway analyses were conducted through comprehensive literature searches using databases such as Human Metabolome Database and Kyoto Encyclopedia of Genes and Genomes (KEGG), as well as by reviewing the metabolic pathways reported within the included articles themselves. These analyses identified common biochemical pathways associated with the significant metabolites.

**FIGURE 2:**
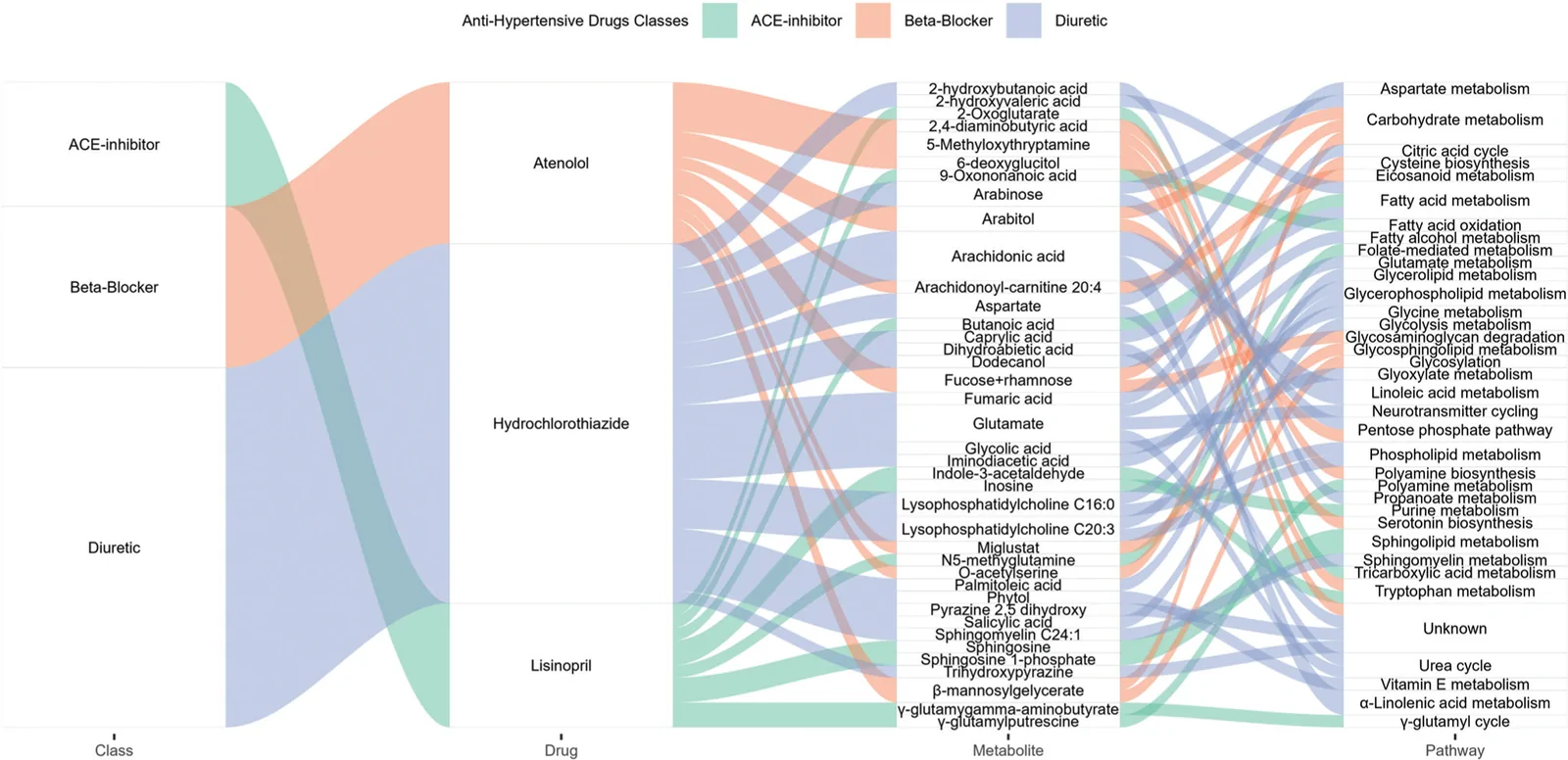
Distribution of identified metabolites across biochemical pathways.!ExcludingUncharacterised Species: IDs 213253, 223548, 21604, 340257, 228377, 470983, 483390.

A pathway analysis was performed of the metabolites identified above for treatment response with diuretics, beta-blockers and ACEi. Several metabolites appear repeatedly across different pathways (Fig. 2). Arachidonic acid, involved in the Eicosanoid metabolism pathway, was identified in studies involving diuretics and beta-blockers.

Metabolites linked to BP responses differed by antihypertensive drug class. For diuretics, metabolites involved in the urea cycle (aspartate, fumaric acid, glutamate) and carbohydrate pathways (arabinose, arabitol) were identified. For beta-blockers, carbohydrate-related metabolites (arabinose, arabitol) and lipid-related pathways (glycerophospholipid and phospholipid metabolism) were associated with treatment response. In ACE inhibitors, metabolites from tryptophan, sphingolipid, and gamma-glutamyl pathways were associated with response. Notably, five fatty acid–related metabolites such as 2-hydroxyvaleric acid, arachidonoyl-carnitine CAR[20 : 4], butanoic acid, caprylic acid, and palmitoleic acid, show significant overlap within the fatty acid metabolism pathway and were correlated with responses to all three drug classes.

## DISCUSSION

### Overview of key metabolomic findings

This systematic review identified several metabolites significantly associated with BP responses to antihypertensive drugs, including 2-oxoglutarate, arachidonoyl-carnitine (CAR 20 : 4), sphingolipids, and selected amino acids. These metabolomic signatures may serve as clinically meaningful biomarkers for predicting therapeutic response in primary hypertension. Notably, several of the metabolites identified map onto pathways connected to drug mechanisms, sympathetic activity, mitochondrial function, and endothelial regulation, suggesting a plausible biological rationale for the observed associations.

#### Tricarboxylic acid cycle intermediates

Multiple studies highlighted 2-oxoglutarate as a consistently replicated biomarker associated with antihypertensive drug response [20,21]. As a key intermediate in the tricarboxylic acid (TCA) cycle, 2-oxoglutarate is involved in the antihypertensive drug classes atenolol and lisinopril [26]. In study by Rotroff *et al.*[21], decreased levels of 2-oxoglutarate predicted lower diastolic BP in response to atenolol. Similarly, Sonn *et al.*[20] demonstrated that elevated baseline 2-oxoglutarate predicted a poorer systolic BP response to lisinopril.

Elevated 2-oxoglutarate has also been linked to metabolic disturbances implicated in the pathogenesis of heart failure, a condition preceded by hypertension in approximately 75% of cases [27]. These findings suggest that dysregulated 2-oxoglutarate metabolism may contribute to the transition from hypertension to heart failure.

In resistant hypertension, higher baseline 2-oxoglutarate and related TCA metabolites such as citrate, oxaloacetate, and malate were characteristic of nonresponders to spironolactone therapy [28]. These findings suggest that baseline 2-oxoglutarate levels may serve as a predictive biomarker to identify patients who are unlikely to respond to lisinopril, potentially enabling clinicians to identify patients unlikely to benefit from lisinopril and to select a more appropriate alternative antihypertensive therapy.

### Arachidonoyl-carnitine and beta-blocker response

Arachidonoyl-carnitine (CAR 20 : 4) emerged as a notable adverse response biomarker for atenolol treatment [24]. Mechanistically, this metabolite reflects alterations in fatty-acid transport into mitochondria via the carnitine–acylcarnitine transporter, enabling mitochondrial β-oxidation of arachidonic acid to acetyl-CoA [29], which in turn feeds into the TCA cycle and links to intermediates such as 2-oxoglutarate.

Atenolol, a cardioselective β-1 adrenergic blocker, reduces SNS activity by inhibiting β-1 adrenergic receptor–mediated inotropic and chronotropic responses [30]. SNS stimulation is closely tied to platelet activation, aggregation, and coagulation processes; catecholamines such as adrenaline can increase platelet size and reactivity [31]. Unlike nonselective agents such as propranolol, atenolol does not significantly inhibit arachidonic acid–induced platelet aggregation or thromboxane formation [32], supporting the concept that CAR 20 : 4-associated effects arise from metabolic, rather than platelet-specific, pathways. Weng *et al.*[24] proposed that pretreatment assessment of CAR 20 : 4 may help identify patients at risk for unfavourable metabolic changes, such as elevated glucose or reduced HDL cholesterol [24] and may thus guide safer beta-blocker prescribing.

### Sphingolipid pathways and angiotensin-converting enzyme inhibitor response

Lisinopril's primary mechanism involves competitive ACE inhibition, reducing conversion of angiotensin I to angiotensin II and lowering aldosterone secretion [33]. ACE inhibition also decreases angiotensin II–mediated negative feedback, increasing renin activity. As ACE degrades bradykinin, inhibition can elevate bradykinin and contribute to angioedema [34].

Sphingolipid metabolism, particularly sphingosine and sphingosine-1-phosphate (S1P), demonstrated consistent associations with favourable BP responses to lisinopril [20]. S1P regulates vascular tone, angiogenesis, endothelial barrier function, and immune cell trafficking [35–38].

Although lisinopril undergoes minimal metabolism and does not directly regulate sphingolipid pathways [39], improvements in endothelial function and reductions in angiotensin II may indirectly modulate sphingosine and S1P signalling. Angiotensin II increases plasma S1P, enhances sphingosine kinase 2 activity, promotes T-cell trafficking, and contributes to microvascular inflammation and hypertension [40]. ACE inhibitors diminish this angiotensin II–S1P axis, thereby reducing immune activation and endothelial dysfunction [41–43].

Comparative clinical observations suggest that ACE inhibitors may produce higher circulating S1P levels than ARBs, indicating potential differences in how these drug classes influence S1P distribution and vascular actions [40,41]. This indirect modulation may explain the positive association between S1P levels and lisinopril responsiveness.

### Metabolomic associations with hydrochlorothiazide

Hydrochlorothiazide (HCTZ) reduces BP through inhibition of the Na^+^/Cl^-^ cotransporter in the early distal convoluted tubule [44,45], leading to natriuresis and reduced plasma volume. Over time, decreased peripheral vascular resistance becomes the dominant antihypertensive mechanism, potentially via activation of calcium-dependent K^+^ channels, inhibition of vascular carbonic anhydrase, and modulation of Rho–ROCK pathways [46–49].

Metabolomic studies have identified baseline aspartate, glutamate, lysophosphatidylcholine C16 : 0, lysophosphatidylcholine C20 : 3, and sphingomyelin C24 : 1 as potential predictors of HCTZ response [23,22]. These metabolites likely reflect broader metabolic or vascular phenotypes rather than components of HCTZ's direct pharmacologic mechanism [50], suggesting their utility lies in prediction rather than mechanistic explanation.

### Methodological considerations and limitations

Quantitative reporting of BP changes attributable to specific metabolites was inconsistent, limiting clinical interpretability. Many included studies were secondary analyses from shared cohorts, particularly the PEAR study [51,52]. Raising concerns regarding selective reporting, population overrepresentation, and limited external validity.

Some metabolites showed inconsistent directionality of association across different studies, drug classes, or populations. These discrepancies may reflect underlying heterogeneity in metabolic states, ethnicity, comorbidities, or methodological differences. It is also plausible that metabolites exert distinct physiological effects depending on the pharmacodynamics of individual antihypertensive drugs.

### Future directions

Future work should broaden metabolomics research to include additional antihypertensive drug classes, particularly mineralocorticoid receptor antagonists and calcium channel blockers, as well as combination therapies and should also prioritize mechanistic validation. Moreover, future studies should extend to investigating these metabolic effects in cases of secondary hypertension. CAR [20 : 4], S1P, and 2-oxoglutarate in particular warrant functional studies to clarify their roles in mitochondrial energetics, endothelial signalling, immune modulation, and hypertension pathophysiology.

Prospective trials incorporating metabolite-guided therapy could enable personalized hypertension treatment strategies, optimizing drug choice and minimizing adverse metabolic consequences.

## CONCLUSION

Metabolomic profiling has identified promising biomarkers, including 2-oxoglutarate, arachidonoyl-carnitine, and sphingolipids, associated with antihypertensive drug responses. Although further validation is required, integrating these biomarkers into clinical practice has potential to enhance personalised treatment strategies for hypertension.

## ACKNOWLEDGEMENTS

None.

7 HYPERMARKER Consortium: A. Acharjee, A. Baak, B. Beger, I. Bos, L. Bravo Merodio, D. Brind, K. Bunting, A. Carter, A. Champsi, M. Chapman, A. Douwes, A. Duarte, M. Elkhalek, D. Engler, A. Ferrer-Albero, P. Gal, C. Gilbert, G. Gkoutos, K. Grijsbach, X. Guan, A. Hogenboom, A. Johnson, M. Jose Forner, B. Lagerwaard, F. Martinez, A. Mcguire, B. Miah, A. Mobley, S. Montes, E. Mossialos, S. Muller, A. Muñoz, A. Nithyanandan, D. Okyere, D. Parkhi, J. Redon, C. Reinbold, G. Sambataro, P. Schlieker, R. Schnabel, E. Schulte, D. Smith, W. Spiering, C. Steinbeisser, A. Uijl, Y. van der Schouw, O. Van Dijk, N. Veerman, R. Vermeulen, J. Vlaanderen, X. Wang, J. Williams, Y. Xu, S. Zeitouny

There are no human participants in this article and informed consent is not required.

The HYPERMARKER project is funded by the European Union under Grant Agreement number 101095480 and United Kingdom Research and Innovation (UKRI). Views and opinions expressed are however those of the author(s) only and do not necessarily reflect those of the European Union or UKRI. Neither the European Union, UKRI nor the granting authorities can be held responsible for them.

### Conflicts of interest

The author(s) declared no potential conflicts of interest with respect to the research, authorship, and/or publication of this article.

## Supplementary Material

Supplemental Digital Content
