# Supplementary material for: Pharmacometabolomics of treatment response to antihypertensive drugs: a systematic review
Source: J Hypertens. 2026 Jun 20;44(9):1477–86. doi: 10.1097/HJH.0000000000004355 (PMC13566420; doi:10.1097/HJH.0000000000004355)
Supplement: Supplementary file 1 [file jhype-44-1477-s001.doc]

Supplementary

**Table S1** Search strategy

((("Metabolome"[MeSH Terms] OR "Metabolomes"[Title/Abstract] OR "Metabolic Profile"[Title/Abstract] OR "Metabolic Profiles"[Title/Abstract] OR "profile metabolic"[Title/Abstract] OR "profiles metabolic"[Title/Abstract] OR "Metabolomic"[Title/Abstract] OR "Metabolomics"[MeSH Terms] OR "Metabonomics"[Title/Abstract] OR "Metabonomic"[Title/Abstract] OR "metabol*"[Title/Abstract] OR

("Lipidomics"[MeSH Terms] OR "lipidom*"[Title/Abstract] OR "Lipid Metabolism"[Title/Abstract] OR "Lipogenesis"[Title/Abstract] OR "Lipid Mobilization"[Title/Abstract] OR "lipol*"[Title/Abstract] OR

"Computational Biology"[MeSH Terms] OR "computational biolog*"[Title/Abstract] OR "computational molecular biolog*"[Title/Abstract] OR "Systems Biology"[Title/Abstract] OR "bio informatic*"[Title/Abstract] OR "Bioinformatic"[Title/Abstract] OR "molecular biolog*"[Title/Abstract] OR "proteomic*"[Title/Abstract] OR "multiomic*"[Title/Abstract] OR

"Molecular Biology"[MeSH Terms] OR "molecular biolog*"[Title/Abstract] OR "Molecular-Medicine"[Title/Abstract] OR "Molecular-Epidemiology"[Title/Abstract] OR

"Mass spectrometry"[MeSH Terms] OR "spectrum analysis mass"[Title/Abstract] OR "analysis mass spectrum"[Title/Abstract] OR "Mass Spectrum Analyses"[Title/Abstract] OR "Mass Spectrum Analysis"[Title/Abstract] OR "spectrometry mass"[Title/Abstract] OR "spectroscopy mass"[Title/Abstract] OR "Gas-Chromatography-Mass-Spectrometry"[Title/Abstract] OR "Liquid-Chromatography-Mass-Spectrometry"[Title/Abstract] OR "GCMS"[Title/Abstract] OR "Gas-Liquid-Chromatography-Mass Spectrometry"[Title/Abstract] OR "Hydrogen Deuterium Exchange-Mass Spectrometry"[Title/Abstract] OR "Chromatography, Liquid"[MeSH Terms] OR "Liquid Chromatography"[Title/Abstract] OR "Chromatography, Reverse-Phase"[ MeSH Terms] OR "Chromatography, Reverse Phase"[Title/Abstract] OR "Reverse-Phase Chromatography"[Title/Abstract] OR "Reverse Phase Chromatography"[Title/Abstract] OR "Reverse-Phase Liquid Chromatography"[Title/Abstract] OR "Reversed-Phase Liquid Chromatography"[Title/Abstract] OR "Chromatography, Reversed-Phase Liquid"[Title/Abstract] OR "Reversed-Phase Chromatography"[Title/Abstract] OR "Chromatography, Reversed-Phase"[Title/Abstract] OR "Spectrometry, Mass, Electrospray Ionization"[Title/Abstract] OR "Spectrometry, Mass, Matrix-Assisted Laser Desorption-Ionization"[MeSH Terms] OR "Chromatography, High Pressure Liquid"[MeSH Terms] OR "HPLC"[Title/Abstract] OR "Chromatography, High Speed Liquid"[Title/Abstract] OR "High-Performance Liquid Chromatography"[Title/Abstract] OR "Chromatography, High-Performance Liquid"[Title/Abstract] OR "High-Performance Liquid Chromatographies"[Title/Abstract] OR "Liquid Chromatography, High-Performance"[Title/Abstract] OR "Chromatography, High Performance Liquid"[Title/Abstract] OR "Tandem Mass Spectrometry"[Title/Abstract] OR "Spectrum Analysis"[MeSH Terms] OR "Spectrometry"[Title/Abstract] OR "Spectroscopy"[Title/Abstract]))

AND

("Blood pressure"[MeSH Terms] OR "Hypertension"[MeSH Terms] OR "Essential Hypertension"[MeSH Terms] OR "High Blood Pressure"[Title/Abstract] OR "High Blood Pressures"[Title/Abstract] OR "blood pressures high"[Title/Abstract] OR "blood pressure high"[Title/Abstract] OR "Primary Hypertension"[Title/Abstract] OR "Diastolic Pressure"[Title/Abstract] OR "pressure diastolic"[Title/Abstract] OR "Pulse Pressure"[Title/Abstract] OR "pressure pulse"[Title/Abstract] OR "Systolic Pressure"[Title/Abstract] OR "pressure systolic"[Title/Abstract] OR "pressures systolic"[Title/Abstract] OR "Isolated Systolic Hypertension"[MeSH Terms] OR "hypertension isolated systolic"[Title/Abstract] OR "systolic hypertension isolated"[Title/Abstract] OR "Systolic Hypertension"[Title/Abstract] OR "hypertension systolic"[Title/Abstract] OR "Hemodynamics"[MeSH Terms] OR "Hemodynamic"[Title/Abstract] OR "Arterial Pressure"[MeSH Terms] OR "Arterial Pressures"[Title/Abstract] OR "pressure arterial"[Title/Abstract] OR "pressures arterial"[Title/Abstract] OR "Arterial Tension"[Title/Abstract] OR "Arterial Tensions"[Title/Abstract] OR "tension arterial"[Title/Abstract] OR "tensions arterial"[Title/Abstract] OR "blood pressure arterial"[Title/Abstract] OR "Arterial Blood Pressure"[Title/Abstract] OR "Arterial Blood Pressures"[Title/Abstract] OR "blood pressures arterial"[Title/Abstract] OR "pressure arterial blood"[Title/Abstract] OR "pressures arterial blood"[Title/Abstract] OR "Aortic Pulse Pressure"[Title/Abstract] OR "Aortic Pulse Pressures"[Title/Abstract] OR "pressure aortic pulse"[Title/Abstract] OR "pressures aortic pulse"[Title/Abstract] OR "pulse pressure aortic"[Title/Abstract] OR "pulse pressures aortic"[Title/Abstract] OR "Mean Arterial Pressure"[Title/Abstract] OR "arterial pressure mean"[Title/Abstract] OR "arterial pressures mean"[Title/Abstract] OR "Mean Arterial Pressures"[Title/Abstract] OR "pressure mean arterial"[Title/Abstract] OR "pressures mean arterial"[Title/Abstract] OR "Aortic Pressure"[Title/Abstract] OR "Aortic Pressures"[Title/Abstract] OR "pressure aortic"[Title/Abstract] OR "pressures aortic"[Title/Abstract] OR "Aortic Tension"[Title/Abstract] OR "Aortic Tensions"[Title/Abstract] OR "tension aortic"[Title/Abstract] OR "tensions aortic"[Title/Abstract] OR "blood pressure aortic"[Title/Abstract] OR "Aortic Blood Pressure"[Title/Abstract] OR "Aortic Blood Pressures"[Title/Abstract] OR "blood pressures aortic"[Title/Abstract] OR "pressure aortic blood"[Title/Abstract] OR "pressures aortic blood"[Title/Abstract] OR "Mean Aortic Pressure"[Title/Abstract] OR "aortic pressure mean"[Title/Abstract] OR "aortic pressures mean"[Title/Abstract] OR "Mean Aortic Pressures"[Title/Abstract] OR "pressure mean aortic"[Title/Abstract] OR "pressures mean aortic"[Title/Abstract])

AND

("Antihypertensive Agents"[MeSH Terms] OR "agents antihypertensive"[Title/Abstract] OR "anti hypertensive agent"[Title/Abstract] OR "anti hypertensive agent"[Title/Abstract] OR "anti hypertensive drug"[Title/Abstract] OR "anti hypertensive drug"[Title/Abstract] OR "drug anti hypertensive"[Title/Abstract] OR "Antihypertensive Drug"[Title/Abstract] OR "drug antihypertensive"[Title/Abstract] OR "Antihypertensives"[Title/Abstract] OR "anti hypertensive agents"[Title/Abstract] OR "anti hypertensive agents"[Title/Abstract] OR "anti hypertensive drugs"[Title/Abstract] OR "anti hypertensive drugs"[Title/Abstract] OR "drugs anti hypertensive"[Title/Abstract] OR "anti hypertensives"[Title/Abstract] OR "anti hypertensives"[Title/Abstract] OR "Antihypertensive Drugs"[Title/Abstract] OR "drugs antihypertensive"[Title/Abstract] OR "anti hypertensive"[Title/Abstract] OR "anti hypertensive"[Title/Abstract] OR "Antihypertensive"[Title/Abstract] OR

("Adrenergic alpha-Antagonists"[MeSH Terms] OR "adrenergic alpha antagonists"[Title/Abstract] OR "adrenergic alpha antagonists"[Pharmacological Action] OR ("adrenergic"[Title/Abstract] AND "alpha antagonists"[Title/Abstract]) OR "alpha adrenergic receptor blockaders"[Title/Abstract] OR ("blockaders"[Title/Abstract] AND "alpha"[Title/Abstract] AND "adrenergic"[Title/Abstract] AND "receptor"[Title/Abstract]) OR "alpha adrenergic blocking agents"[Title/Abstract] OR "agents alpha adrenergic blocking"[Title/Abstract] OR "blocking agents alpha adrenergic"[Title/Abstract] OR "alpha adrenergic blockers"[Title/Abstract] OR "alpha adrenergic antagonists"[Title/Abstract] OR "adrenergic alpha receptor blockaders"[Title/Abstract] OR "adrenergic alpha 1 receptor antagonists"[Title/Abstract] OR "adrenergic alpha1 antagonists"[Title/Abstract] OR "alpha 1 adrenergic blocking agents"[Title/Abstract] OR "alpha1 adrenoceptor blocker"[Title/Abstract] OR "adrenergic alpha 2 receptor antagonists"[Title/Abstract] OR "alpha 2 adrenergic blocking agents"[Title/Abstract] OR "adrenergic alpha2 antagonists"[Title/Abstract] OR "alpha2 adrenergic antagonists"[Title/Abstract] OR

"Adrenergic beta-Antagonists"[MeSH Terms] OR "adrenergic beta antagonists"[Title/Abstract] OR "beta antagonists adrenergic"[Title/Abstract] OR "adrenergic beta receptor blockader"[Title/Abstract] OR "beta adrenergic antagonist"[Title/Abstract] OR "beta adrenergic blocker"[Title/Abstract] OR "beta adrenergic antagonists"[Title/Abstract] OR "beta adrenoceptor antagonists"[Title/Abstract] OR "adrenergic beta receptor blockaders"[Title/Abstract] OR "beta adrenergic receptor blockaders"[Title/Abstract] OR "beta adrenergic blocking agents"[Title/Abstract] OR "agents beta adrenergic blocking"[Title/Abstract] OR "beta adrenergic blockers"[Title/Abstract] OR "blockers beta adrenergic"[Title/Abstract] OR "Adrenergic beta-Blockers"[Title/Abstract] OR "beta adrenergic blocking agent"[Title/Abstract] OR "beta adrenergic receptor blockader"[Title/Abstract] OR "beta adrenoceptor antagonist"[Title/Abstract] OR "adrenergic beta antagonist"[Title/Abstract]) OR

((((("angiotensin-converting enzyme inhibitors"[MeSH Terms] OR "angiotensin converting enzyme inhibitors"[Title/Abstract] OR "enzyme inhibitors angiotensin converting"[Title/Abstract] OR "inhibitors angiotensin converting enzyme"[Title/Abstract] OR "angiotensin converting enzyme inhibitors"[Pharmacological Action] OR ("angiotensin converting"[Title/Abstract] AND "enzyme"[Title/Abstract] AND "inhibitors"[Title/Abstract]) OR "inhibitors"[Title/Abstract]) AND "kininase"[Title/Abstract] AND "ii"[Title/Abstract]) OR "kininase II Inhibitors"[Title/Abstract] OR "angiotensin converting enzyme antagonists"[Title/Abstract] OR "Kininase II Inhibitor"[Title/Abstract] OR "ii inhibitor kininase"[Title/Abstract] OR "inhibitor kininase ii"[Title/Abstract] OR "enzyme"[Title/Abstract]) AND "antagonists"[Title/Abstract] AND "angiotensin"[Title/Abstract] AND "converting"[Title/Abstract]) OR "inhibitors ace"[Title/Abstract] OR "ACE Inhibitors"[Title/Abstract] OR "angiotensin i converting enzyme inhibitors"[Title/Abstract] OR "angiotensin converting enzyme inhibitor"[Title/Abstract] OR "ACE Inhibitor"[Title/Abstract] OR "inhibitor ace"[Title/Abstract] OR "angiotensin i converting enzyme inhibitor"[Title/Abstract] OR "enzyme inhibitor angiotensin converting"[Title/Abstract] OR "inhibitor angiotensin converting enzyme"[Title/Abstract] OR "antagonists angiotensin converting enzyme"[Title/Abstract]) OR

"Diuretics"[MeSH Terms] OR "Diuretic"[Title/Abstract] OR "Diuretic Effect"[Title/Abstract] OR "effect diuretic"[Title/Abstract] OR "Diuretic Effects"[Title/Abstract] OR "effects diuretic"[Title/Abstract] OR

"Vasodilator Agents"[MeSH Terms] OR "agents vasodilator"[Title/Abstract] OR "Vasodilators"[Title/Abstract] OR "Vasorelaxant"[Title/Abstract] OR "Vasodilator"[Title/Abstract] OR "Vasodilator Agent"[Title/Abstract] OR "agent vasodilator"[Title/Abstract] OR "Vasodilator Drug"[Title/Abstract] OR "drug vasodilator"[Title/Abstract] OR "Vasodilator Drugs"[Title/Abstract] OR "drugs vasodilator"[Title/Abstract] OR "Vasorelaxants"[Title/Abstract] OR "Vasoactive Antagonists"[Title/Abstract])) OR

"Angiotensin Receptor Antagonists"[MeSH Terms] OR "Antagonists, Angiotensin Receptor"[Title/Abstract] OR "Angiotensin Receptor Blockers"[Title/Abstract] OR "Angiotensin Receptor Antagonist"[Title/Abstract] OR "Angiotensin II Receptor Antagonists"[Title/Abstract] OR "Angiotensin II Receptor Antagonist"[Title/Abstract] OR "Angiotensin II Receptor Blockers"[Title/Abstract] OR "Angiotensin II Receptor Blocker"[Title/Abstract] OR "Angiotensin II Type 1 Receptor Blockers"[Title/Abstract] OR "Angiotensin II Type 1 Receptor Blocker"[Title/Abstract] OR "Angiotensin II Type 2 Receptor Blockers"[Title/Abstract] OR "Angiotensin II Type 2 Receptor Blockers"[Title/Abstract] OR "Angiotensin 2 Type 1 Receptor Antagonists"[Title/Abstract]) OR

("Calcium Channel Blockers"[MeSH Terms] OR "Calcium Channel Blocking Drug"[Title/Abstract] OR "channel blockers calcium"[Title/Abstract] OR "Calcium Channel Antagonists"[Title/Abstract] OR "antagonists calcium channel"[Title/Abstract] OR "Calcium Channel Blocking Drugs"[Title/Abstract] OR "Calcium Channel Antagonist"[Title/Abstract] OR "antagonist calcium channel"[Title/Abstract] OR "channel antagonist calcium"[Title/Abstract] OR "Calcium Channel Blocker"[Title/Abstract] OR "blocker calcium channel"[Title/Abstract] OR "channel blocker calcium"[Title/Abstract])

AND "english"[Language] AND (("observational study"[Publication Type] OR "randomised controlled trial"[Publication Type]) AND "humans"[MeSH Terms] AND "english"[Language]) AND (("clinical trial"[Publication Type] OR "observational study"[Publication Type] OR "randomised controlled trial"[Publication Type]) AND "humans"[MeSH Terms] AND "english"[Language]) AND (("clinical trial"[Publication Type] OR "observational study"[Publication Type] OR "randomised controlled trial"[Publication Type]) AND "humans"[MeSH Terms] AND "english"[Language]) AND (("clinical trial"[Publication Type] OR "observational study"[Publication Type] OR "randomised controlled trial"[Publication Type]) AND "humans"[MeSH Terms] AND "english"[Language]) AND (("clinical trial"[Publication Type] OR "observational study"[Publication Type] OR "randomised controlled trial"[Publication Type]) AND "humans"[MeSH Terms] AND "english"[Language]) AND (("clinical trial"[Publication Type] OR "observational study"[Publication Type] OR "randomised controlled trial"[Publication Type]) AND "humans"[MeSH Terms] AND "english"[Language]) AND ((y_10[Filter]) AND (clinicaltrial[Filter] OR observationalstudy[Filter] OR randomisedcontrolledtrial[Filter]) AND (humans[Filter]) AND (english[Filter]) AND (alladult[Filter]))

**Table S2 Summary of Clinical Trials Evaluating Different Antihypertensive Drug Classes Across Hypertension Patient Populations**

| **First Author** | **Drug class** | **Blood pressure (mmHg)** | **Mean BP Change (mmHg)** | **Patient Population** | **Wash out period** | **Treatment**  **period** | **Daily Dose** | **Age criteria** | **Cohort (Country)** |
| --- | --- | --- | --- | --- | --- | --- | --- | --- | --- |
| Shahin *et al.* | Diuretics | >120/70 | SBP: −11.4 /DBP: −6.9 (C-allele carriers),  SBP/DBP: −6.8/−3.5 (non-carriers) | White n=123 | 3 weeks | 3-9 weeks | 12,5mg or 25mg | 17-65 | PEAR^1^ (US) |
| Rotroff *et al.* | Diuretics | >120/70 | Home DBP  -4.5±0.51 | White  n =123 | Min 18 days | 3-9 weeks | 12,5mg or 25mg | 17-65 | PEAR (US) |
| Rotroff *et al.* | Diuretics | >120/70 | Home DBP  -6.8±0.73 | Black n=83 | Min 18 days | 3-9 weeks | 12,5mg or 25mg | 17-65 | PEAR (US) |
| Huang *et al.* | Diuretics | >140/90 | Office SBP −18.6± 15.1 /DBP  −10.2 ± 8.9 | Asian n=30 | 2 weeks | 2 weeks | 50mg | >25 | T-VGH (Taiwan) |
| Rotroff *et al.* | Beta - Blocker | >120/70 | Home DBP  -4.0±0.64 | Black n=109 | Min 18 days | 3-9 weeks | 50mg or 100mg | 17-65 | PEAR (US) |
| Rotroff *et al.* | Beta - Blocker | >120/70 | Home DBP  -10.13±0.6 | White n=128 | Min 18 days | 3-9 weeks | 50mg or 100mg | 17-65 | PEAR (US) |
| Weng *et al.* | Beta - Blocker | >120/70 | SBP  −12.7 ± 8.6 /DBP  −10.4 ± 5.8 | White n=224 | 3-8 weeks | 3-9 weeks | 50mg or 100mg | 17-65 | PEAR (US) |
| Sonn *et al.* | ACEi | >140/90 | MAP^2^  Responders: −18.9; non-responders: −1.45 mmHg | White n=45 |  | 1 week | 10mg | 18-89 | PPME (US) |

^1PEAR: Pharmacogenomics Evaluation of Antihypertensive Response study (52); PPME: Pharmacogenetic Prediction of Metoprolol; 2 MAP:^ ^Mean arterial pressure^

^Effectiveness(51) T-VGH: Taipei Veterans general hospital study number 2011-07-010GB and 2019-02-00^
